# Supplementary material for: Activation of calcineurin underlies altered trafficking of α2 subunit containing GABAA receptors during prolonged epileptiform activity
Source: Neuropharmacology. 2015 Jan;88:82–90. doi: 10.1016/j.neuropharm.2014.09.014 (PMC4239296; doi:10.1016/j.neuropharm.2014.09.014)
Supplement: Supplementary file 1 [file mmc1.docx]

1. **Introduction**

GABA_A_ Receptors (GABA_A_Rs) are ligand-gated chloride permeable ion channels which mediate both phasic (synaptic) and tonic (extrasynaptic) inhibitory neurotransmission in the central nervous system (Jacob et al. 2008; Luscher et al. 2011). They assemble from five subunits, the composition of which determines the receptors functional and pharmacological properties and the specific location on the neuronal membrane (Luscher et al. 2011; Jacob et al. 2008). GABA_A_Rs containing the γ2 subunit mediate synaptic transmission (in contrast to extrasynaptic receptors located away from the synapse) and are a target for benzodiazepines (Pritchett et al. 1989). The enrichment of GABA_A_Rs in subcellular compartments such as the axon initial segment (AIS) has been reported for the α2 subunit and although both α1 and α2 subunits are found at the synapse in dendrites, a minority of GABA_A_Rs in the AIS contain the α1 subunit (Panzanelli et al. 2011; Brünig et al. 2002). GABA_A_Rs undergo dynamic movement within the cellular membrane. Lateral diffusion facilitates trafficking and assures the appropriate surface localisation of the receptor (Mukherjee et al. 2011), while trafficking to and from the membrane through exocytotic and endocytotic processes allows constant maintenance of the inhibitory synaptic receptor pool (Bogdanov et al. 2006; Kittler et al. 2000; Kittler et al. 2004). Altered neuronal activity causes surface GABA_A_Rs to undergo plasticity-induced trafficking changes. These are mediated by alterations in the activity of protein phosphatases and kinases and linked to increased intracellular Ca^2+^ changes (Muir et al. 2010; Bannai et al. 2009; Saliba et al. 2012; Luscher et al. 2011; Jacob et al. 2008; Petrini et al. 2014).

SE evolves rapidly and dynamically, manifesting as a prolonged and self-sustaining seizure with significant morbidity and mortality (Lothman 1990; Dodrill & Wilensky 1990; Sutter et al. 2013). This distinct condition can occur in patients with previous epilepsy or may occur de novo as a result of acute neurological disorders (Trinka et al. 2012). As SE evolves, the patient’s response to treatment with benzodiazepines decreases progressively which rapidly results in benzodiazepine pharmacoresistance. This may lead to refractory SE, a pathological state in which seizures are not sopped by first- or second-line anticonvulsant therapies.

To unravel the role of benzodiazepine pharmacoresistance associated with SE patients, studies have addressed whether the trafficking of GABA_A_Rs to and from the cellular membrane is altered during models of SE (Naylor et al. 2005; Goodkin et al. 2005; Blair et al. 2004). Interestingly, it has been suggested that GABA_A_Rs are subjected to subunit-specific trafficking during prolonged depolarisation. GABA_A_Rs containing the synaptic subunits β2/3 and γ2 undergo internalisation whereas the extrasynaptic δ subunit remains unchanged (Goodkin et al. 2008). Despite recent studies, the temporal dynamics of GABA_A_R trafficking have not been investigated using live-cell imaging. Moreover, whether endocytosis occurs preferentially in distinct compartments such as dendrites or soma remains unclear. It is not known which molecular pathways underlie this subunit-specific trafficking of GABA_A_Rs. Furthermore, it remains to be determined whether Ca^2+^ and its intracellular signalling cascades play a significant role in the modulation of GABAergic inhibition during SE.

To address the molecular mechanisms underlying altered GABA_A_R trafficking during SE, we used a live-cell imaging approach to examine the surface stability of GABA_A_Rs in hippocampal neurons. We induced prolonged epileptiform bursting activity *in vitro* by exposing neurons to artificial cerebrospinal fluid (aCSF) lacking Mg^2+^ (Mangan & Kapur 2004; Sombati & Delorenzo 1995). Using this model, we show a decrease in somatic surface GABA_A_Rs that is dependent on NMDAR activity and the Ca^2+^-dependent phosphatase, calcineurin. Furthermore, we show that epileptiform activity alters intracellular Ca^2+^ concentrations, which correlates with the decrease of GABA_A_Rs from the surface possibly contributing to pathological signalling during SE.

1. **Materials and Methods**
   1. **Constructs.**

The N-terminally tagged GABA_A_ α_2_-_SEP_ DNA was a kind gift from S. Moss (Tufts University, Cambridge, MA) and has been described previously (Tretter et al. 2008).

- 1. **Cell Culture and Transfection**.

All animal experiments were carried out in accordance with the U.K. Animals (Scientific Procedures) Act, 1986. All efforts were made to minimise animal suffering and to reduce the number of animals used. Dissected hippocampi of P0 rat pups or E18 embryos were immediately placed in ice-cold dissection buffer (HBSS (Invitrogen)) and washed once. Using trypsin (0.25%) tissue was digested for 10 min before trituration in ~2 ml of attachment medium. Neurons were plated onto poly-L-lysine (Sigma) coated coverslips (500 µg/ml). For nucleofection, hippocampal neurons were nucleofected with GABA_A_ α_2SEP_ plasmid DNA. Neurons were centrifuged and the cell pellet was resuspended in 100 µl transfection buffer (135 mM KCL, 10 mM HEPES-pH 7.3, 2 mM MgCl_2_, 5 mM EGTA, 0.2 mM CaCl_2_) and transfected using a single cuvette AMAXA system (Lonza, programme O-003 or AK-009). Neurons were left to develop at 37°C and 95 % O_2_, 5 % CO_2_ in maintenance medium [Neurobasal (Invitrogen), B27 Supplement (Invitrogen), 0.6 % Glucose (Sigma), 2 mM Glutamine (Invitrogen) and Penicillin-Streptomycin] for 14-21 DIV before imaging.

- 1. **Live-cell Imaging.**

Live-cell Imaging was performed on an upright Olympus microscope (BX51WI) coupled to an EM-CCD camera (Ixon, Andor). Cells were imaged with a water-immersion 60X objective (Olympus). Excitation was provided by an X-cite 120Q light source (Lumen Dynamics). Appropriate filters were used (in nm): Excitation: 470/40; Emission: 525/50; Dichroic: 495, long pass. The image pixel scale was calculated by dividing the camera pixel size (16 μm) by the lens magnification (60X) yielding a pixel size of 0.27 μm. Before constant perfusion with a Cole-Parmer Master-Flex pump (~ 4 ml/min), aCSF (126 mM NaCl, 24 mM NaHCO_3_, 10 mM D-Glucose, 2.5 mM KCL, 2 mM CaCl_2_, 1 mM MgCl, 1 mM NaH_2_PO_4_, 5 mM Sodium Pyruvate) was pre-equilibrated for 20 min with 95 % O_2_ and 5 % CO_2_ to establish a pH of 7.4. Temperature of the waterbath was constantly measured using a digital Thermometer (Hanna Instruments) and maintained at 37°C. Any focus drift was corrected manually. Protocols were adapted to achieve minimal bleaching conditions. Imaging of SEP-tagged GABA_A_Rs was done for 60 min at a rate of one frame per 20 seconds (180 frames, 48.8 ms exposure, no averaging). For imaging of intracellular Ca^2+^ using fluo4 (1 µM, Molecular Probes, Invitrogen) hippocampal neurons were incubated for 30 min at 37 °C. After washing twice, fluo4-imaging was done for 60 min (720 frames, 5 ms exposure, no averaging) at 1 frame every 5 seconds.

- 1. **Cell-attached recording**

Cell-attached recordings were made on transfected hippocampal neurons at 13 DIV using an Axopatch 200B amplifier (Molecular Devices) and pClamp software. Cells were visualised using an upright Olympus BX50WI microscope equipped with a 40x water-immersion objective and infrared optics. Recording electrodes were pulled from standard-walled borosilicate glass capillaries (Warner Instruments) and filled with aCSF. Gigaseal cell attached recordings were made in voltage-clamp mode at -70mV; the cells were constantly perfused with aCSF. To block currents during recording NBQX disodium salt (20 µM, Abcam) and dAPV (D-(-)-2-Amino-5-phosphonopentanoic acid, 25 µM, TOCRIS) were added to the perfusion solution.

- 1. **Low Mg^2+^ and Drug Treatments.**

To induce epileptiform bursting activity, aCSF without Mg^2+^ but 2 μM glycine (126 mM NaCl, 24 mM NaHCO_3_, 10 mM D-Glucose, 2.5 mM KCL, 2 mM CaCl_2_, 0 mM MgCl, 1 mM NaH_2_PO_4_, 5 mM sodium pyruvate, 2µM glycine) was used (Blair et al. 2004). We confirmed previous studies (Robinson et al. 1993; Mangan & Kapur 2004; DeLorenzo et al. 1998) that low Mg^2+^ results in cellular burst spiking that is dependent upon glutamateric transmission (Sup. Fig.2). Moreover, action potentials were associated with post-synaptic currents, indicating that the bursting was the result of network activity (Sup. Fig. 2). The mitochondrial complex I substrate sodium pyruvate was supplemented to reduce neuronal death (Kovac et al. 2012). Transfected hippocampal neurons were perfused with control aCSF for 3.3 min (10 frames, baseline), followed by either low Mg^2+^ treatment or continued perfusion with aCSF with Mg^2+^ only (control) for 60 min. To block NMDAR activity during low Mg^2+^ treatment, the NMDAR blocker dAPV (25 µM, TOCRIS) was used continuously throughout the low Mg^2+^ treatment without preincubation. For low Mg^2+^/NMDA treatment, NMDA (30 µM, TOCRIS) was added to the low Mg^2+^ medium and applied continuously for 60 min. To block the activity of the Ca^2+^ dependent phosphatase calcineurin, cells were pre-incubated with calcineurin autoinhibitory peptide (Terada et al. 2003) (50 µM, Calbiochem) for 25 min at 37 °C and imaged (without application of the peptide during perfusion) either during control or low Mg^2+^ treatment (Muir et al. 2010).

- 1. **Image Analysis.**

Intensity analysis of specific regions of interests (ROIs: background, soma, diffuse, clustered) was done in ImageJ 1.43u which allowed the export of raw data to MatlabR2008a Software. Image correction was done in Image J software using the plugin StackReg macro (Thevenaz et. al. 1998) which corrects for drift in (x,y). Inverted average intensity projection was done in ImageJ by using the Z-stack application from frame 1-10 (0-3 min) and frame 30-60 (10-20 min). Analysis of SEP-imaging raw data was done using Matlab Software through a custom designed code. Background was subtracted from each frame, fluorescence intensity was normalised to the baseline (average value of t = 0-3.33 min) and averaged for each experimental group. The fluorescence intensity values for each specific ROI were analysed in individual loops which allowed separate analysis. Furthermore, the standard error of mean (SEM) was calculated for each time-point and the mean normalised values including error bars were plotted against time. Ca^2+^ imaging was analysed using ImageJ. Fluorescence intensity in the soma was extracted from one ROI per cell. Baseline for 60 min Ca^2+^-imaging was the average of the first 10 frames (t = 0-50 sec), which corresponds to control conditions.

- 1. **Statistical Analysis.**

All experiments were performed on neurons from at least three individual preparations. The software GraphPad Prism was used for statistical tests and to generate bar charts. Data sets were tested to determine if they were normally distributed (KS normality test) before undertaking further statistical analysis. For low Mg^2+^ only and low Mg^2+^ NMDA treatments, p values were determined using a Student’s t test (two-tailed). Repeat measures ANOVA (for normally distributed data) or Friedman test was used to analyse significance of low Mg^2+^ induced effects during Ca^2+^-imaging, low Mg^2+^/dAPV and low Mg^2+^/CAIP experiments since there were more than two experimental groups. Appropriate post-hoc tests such as Tukey’s for normally distributed data or Dunn’s multiple comparison for non-normally distributed data were used. Values are given as mean ± SEM. Error bars represent SEM.

1. **Results**
   1. **Surface Stability of Somatic GABA_A_Rs in Hippocampal Neurons is altered** **during Low Mg^2+^ Treatment.**

To examine the influence of SE on GABA_A_R stability and clustering *in vitro,* we mimicked the characteristic repetitive epileptiform bursting activity of SE by removal of Mg^2+^ from the extracellular medium of cultured hippocampal rat neurons transfected with SEP-tagged GABA_A_R α_2_ subunit (α_2SEP_) (Sombati & Delorenzo 1995). Surface GABA_A_Rs were imaged via the SEP-tag on the α_2_ subunit (Muir et al. 2010) (which allows visualisation through high fluorescence in neutral pH, Sup. Fig.1) for 60 min. α_2SEP_ fluorescence was analysed in 3 distinct regions of interests (ROIs): soma, diffuse (extrasynaptic compartment in dendrites) and clusters. At 20 min, somatic fluorescence of α_2SEP_-containing GABA_A_Rs was significantly decreased (control F/F_0_: 0.997 ± 0.02, low Mg^2+^ F/F_0_: 0.85 ± 0.05; p = 0.02) indicating that internalisation of GABA_A_Rs at the somatic level increases during low Mg^2+^ treatment (Fig.1 C,F). This could account for a decrease in hippocampal GABAergic inhibition during epileptiform activity. However, α_2SEP-_fluorescence intensity at the soma was not found to be significantly changed at t = 60 min (control F/F_0_: 0.92 ± 0.03, low Mg^2+^ F/F_0_: 1.01 ± 0.04; p = 0.09) suggesting a biphasic regulation of surface GABA_A_Rs during low Mg^2+^ treatment (Fig.1 C,F’). Interestingly, α_2SEP_-GABA_A_R clusters (t = 20 min; control F/F_0_: 1.02 ± 0.02 low Mg^2+^ F/F_0_: 0.96 ± 0.07; p = 0.36) and diffuse (t = 20 min; control F/F_0_: 1.01 ± 0.02, low Mg^2+^ F/F_0_: 0.95 ± 0.05; p = 0.28) fluorescence intensity in the neuronal dendrites during low Mg^2+^ treatment showed only a minor, non-significant decrease. Our data thus indicates compartmental specificity of low Mg^2+^ induced decrease of GABA_A_Rs from the surface (Fig.1 D,E), with GABA_A_Rs primarily endocytosed from the cell soma surface.


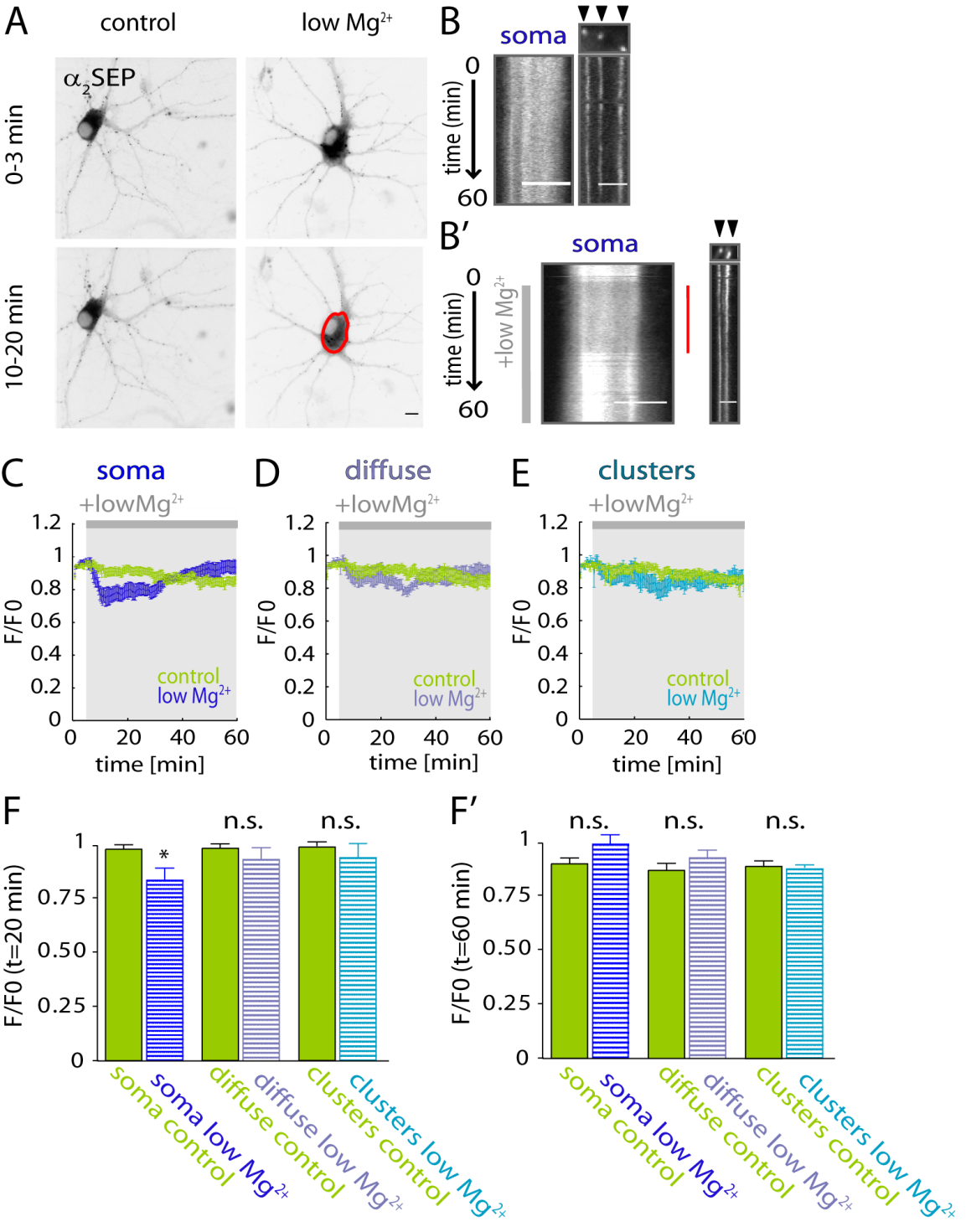


**Figure 1:** Somatic surface α_2SEP_-GABA_A_Rs decrease upon low Mg^2+^ treatment. (A) Representative average intensity projection of α_2SEP_ expression in control and low Mg^2+^ treated neurons over time (0-3 min and 10-20 min). Scale bar, 10 μm. (B) Kymograph (a line scan vertically projected over time) showing somatic (left; scale bar, 5 μm) and clustered (right; arrow heads indicate clusters; scale bar, 2μm) α_2SEP_ fluorescence intensity over the movie in control (aCSF) conditions and in the presence of low Mg^2+^ (grey bar). Red bar on the right indicates a decrease in somatic fluorescence intensity upon low Mg^2+^ treatment. (C) Average fluorescence intensity of somatic α_2SEP_ GABA_A_R F/F0: control (green, n = 9 cells) and low Mg^2+^, (blue, n = 7). (D) Time course of diffuse α_2SEP_ GABA_A_R F/F0: control (green, n = 9 cells); low Mg^2+^, (magenta, n = 7). (E) Time course of α_2SEP_ GABA_A_R clusters F/F0: control (green, n = 9 cells) and low Mg^2+^ ([light](http://www.dict.cc/englisch-deutsch/turquoise.html) blue, n = 7). (F) Bar graph of ROI’s F/F0: soma (left), diffuse (middle) clusters (right). Significant loss of fluorescence in the soma compared to control at 20 min following low Mg^2+^ treatment (p = 0.02). Diffuse fluorescence is not altered at 20 min (p = 0.36) after low Mg^2+^ treatment. Fluorescence intensity of α_2SEP_ GABA_A_R clusters is unaltered following low Mg^2+^ treatment (t = 20 min; p = 0.28) compared to control. (F’) At 60 min after low Mg^2+^ treatment somatic (p = 0.09), diffuse (p = 0.85) and clustered (p = 0.42) fluorescence intensity are not significantly altered.*p < 0.05.

- 1. **Activity of NMDA Receptors Induces the Down-Regulation of Somatic GABA_A_ Receptors from the Surface during Low Mg^2+^ Treatment.**

Low extracellular Mg^2+^ induces epileptiform activity which is abolished by application of the NMDAR antagonist dAPV (Coan & Collingridge 1985; Coan & Collingridge 1987; Tancredi et al. 1990; Albowitz et al. 1997; Westerhoff et al. 1995; Gulyás-Kovács et al. 2002b; Mangan & Kapur 2004). Therefore, we tested whether inhibition of NMDAR activity during low Mg^2+^ treatment blocks the somatic down-regulation of GABA_A_Rs (Fig.2). Low Mg^2+^ alone induced a significant decrease of α_2SEP_-GABA_A_R fluorescence intensity at t = 20 min whereas this loss was inhibited by the co-application of dAPV (t = 20 min; control F/F_0_: 0.97 ± 0.05, low Mg^2+^ F/F_0_: 0.65 ± 0.03 (p < 0.001), low Mg^2+^/dAPV F/F_0_: 0.96 ± 0.07 (p > 0.05); one-way ANOVA test, Tukey’s multiple comparison post test), confirming that the down regulation of surface GABA_A_Rs was dependent on the activation of NMDARs (Fig.2 C,C’).

**
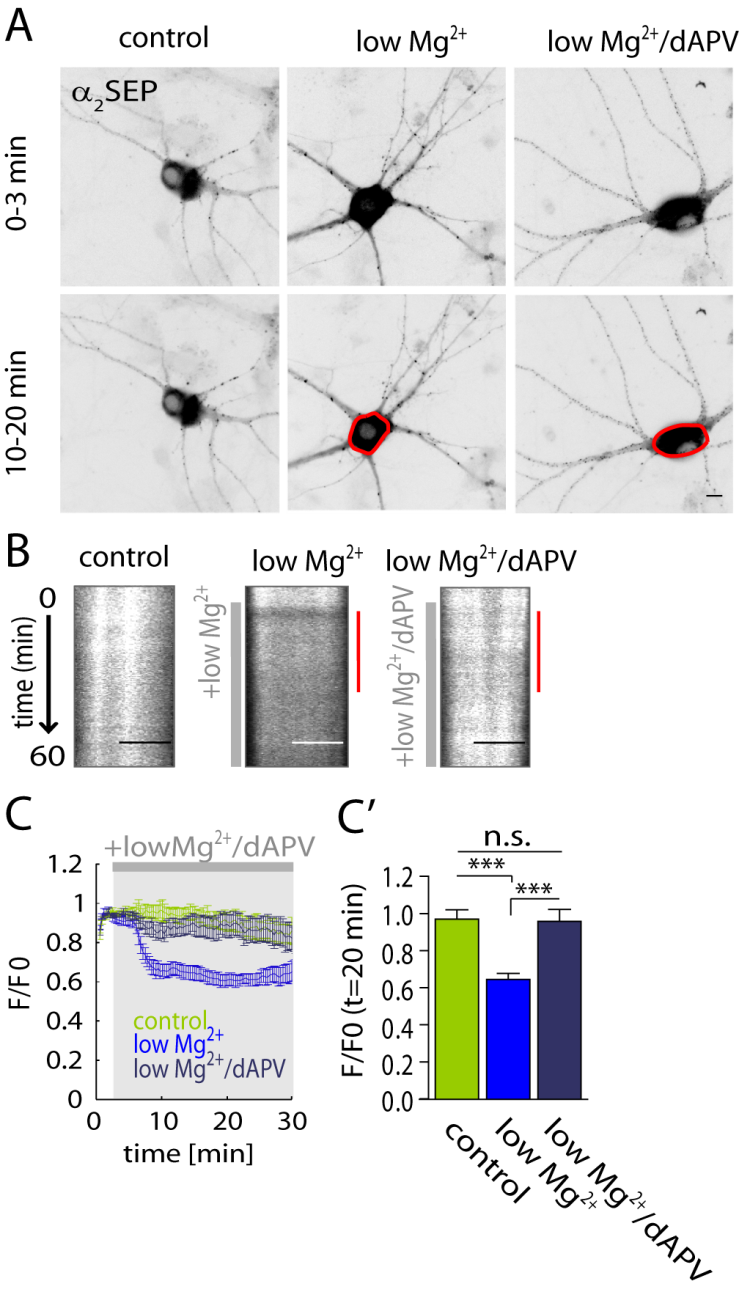
**

**Figure 2:** NMDARs mediate low Mg^2+^ induced somatic α_2SEP_ GABA_A_R surface decrease. (A) Representative images of α_2SEP_ GABA_A_R fluorescence in control, low Mg^2+^ and low Mg^2+^ with dAPV (low Mg^2+^/dAPV) treated neurons as an average intensity projection over time (0-3 min and 10-20 min). Somatic α_2SEP_ GABA_A_R loss highlighted in red; scale bar, 10 μm. (B) Kymograph showing somatic (left; scale bar, 5 μm) fluorescence intensity over the movie (duration: 60 min) in control (aCSF) conditions and in the presence of low Mg^2+^ and low Mg^2+^/dAPV (grey bar). Red bar on the right indicates decrease in somatic fluorescence intensity upon low Mg^2+^ treatment and blocking of low Mg^2+^ induced effect by dAPV (B). (C) Time course of somatic α_2SEP_ GABA_A_R F/F0: control (green, n = 7 cells), low Mg^2+^ (blue, n = 8 cells) and low Mg^2+^/dAPV (dark blue, n = 6 cells). (C’) Summary of somatic F/F0 at 20 min after low Mg^2+^ treatment. Low Mg^2+^ induces a significant decrease (p < 0.001) in somatic fluorescence intensity, which is inhibited by application of NMDAR blocker dAPV (p < 0.001). ***p < 0.001.

- 1. **Epileptiform Activity Evokes Intracellular Ca^2+^ Changes that Correspond to the Temporal Dynamics of Somatic Surface GABA_A_R Decrease.**

To further explore the mechanisms of NMDAR-driven decrease in surface GABA_A_Rs during low Mg^2+^ treatment, we applied the fluorescent Ca^2+^ indicator fluo4 in low Mg^2+^ treated hippocampal neurons. This allowed us to investigate intracellular Ca^2+^ transients evoked by low Mg^2+^ treatment. Hippocampal neurons perfused with control aCSF exhibit small Ca^2+^ transients reflecting spontaneous activity, whereas low Mg^2+^ perfusion significantly altered intracellular Ca^2+^ throughout the timeline of 60 min (Fig.3 B,B’). Fluo4 imaging reported intracellular Ca^2+^ increases rapidly upon early perfusion with low Mg^2+^ (10-20 min F/F0: 375.8 ± 28.4; p < 0.001, Friedman test and Dunn’s multiple comparison post test) and at 60 min (F/F0: 215.2 ± 28.8; p < 0.05, Friedman test and Dunn’s multiple comparison post test) in comparison to baseline (t = 100-150 sec; F/F0: 104.5 ± 4.4), (Fig.3 C). This indicates that low Mg^2+^ treatment induces an intracellular Ca^2+^ rise, which is likely to be caused by activation of NMDARs. Interestingly, intracellular Ca^2+^ concentration drops significantly during the timeline (10-20 min, F/F0: 375.8 ± 28.4; 50-60 min, F/F0: 215.2 ± 28.8; p < 0.05, Friedman test and Dunn’s multiple comparison post test) showing that intracellular Ca^2+^ concentration undergoes alteration on a similar timescale to that of somatic surface GABA_A_R decrease (Fig.3 C).


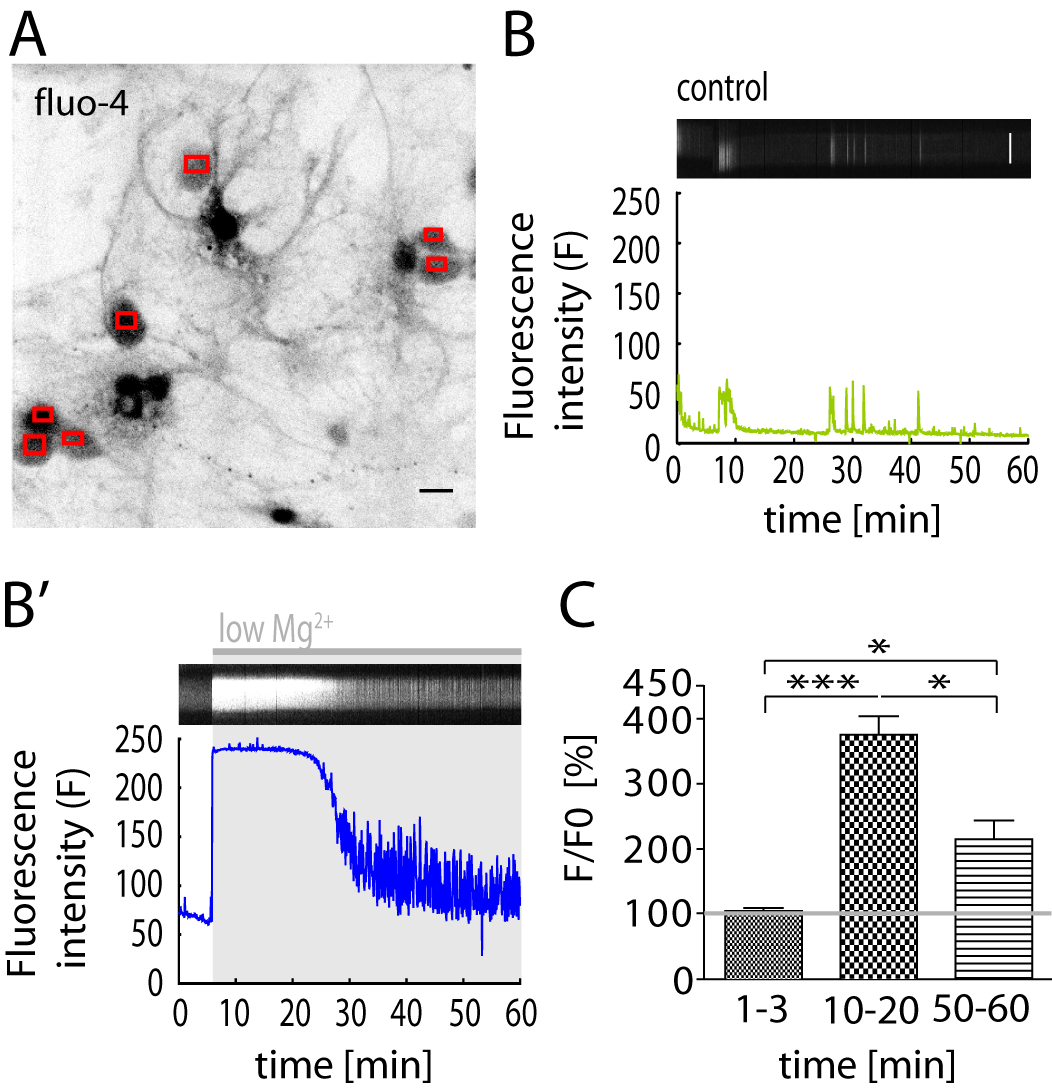


**Figure 3:** Low Mg^2+^ treatment induces intracellular Ca^2+^ accumulation. (A) Representative average intensity projection of fluo-4 loaded neurons (coloured squares indicate individual cell bodies). (B) Raw fluorescence intensity of spontaneous Ca^2+^ transients in a neuron (bottom) and correlating Kymograph (top; segmented line through somatic ROI) showing fluorescence changes over time under control (aCSF) conditions. (B’) Raw fluorescence intensity reporting low Mg^2+^ (grey bar) induced Ca^2+^ accumulation (bottom) and Kymograph (top) indicating increase in fluorescence intensity. (C) Quantification of increase in intracellular Ca^2+^ (n = 21 cells). Between 10-20 min (averaged data points from min 10 to min 20) after low Mg^2+^ induction, intracellular Ca^2+^ is significantly elevated (p < 0.001) compared to baseline (averaged data points from min 0 to min 3). At 50-60 min intracellular Ca^2+^ has decreased (p < 0.001) compared to 10-20 min and significantly increased (p = 0.02) compared to the baseline. *p < 0.05, ***p < 0.001. Scalebar, 10 µm.

- 1. **Dispersion of Clustered GABA_A_ Receptors is Induced by NMDA Receptor Activation.**

It has been reported that the dispersal of surface GABA_A_R clusters in neuronal processes is regulated through Ca^2+^ influx via NMDARs (Muir et al. 2010). Therefore we tested whether further increasing the activation of NMDARs by co-application of low Mg^2+^ and the agonist NMDA would trigger dispersion of surface GABA_A_R clusters in proximal dendrites. Indeed, the activation of NMDARs with low Mg^2+^ in addition to application of the agonist NMDA (low Mg^2+^/NMDA) caused a loss of α_2SEP_ GABA_A_R fluorescence intensity in dendritic clusters (Fig.4) at t = 20 min (control F/F_0_: 1.001 ± 0.04, low Mg^2+^/NMDA F/F_0_: 0.799 ± 0.03; p < 0.01) suggesting that surface stability of GABA_A_Rs corresponds with the potency of NMDAR activation (Fig.4 C,F). Interestingly, during low Mg^2+^/NMDA perfusion diffuse (control F/F_0_: 1.02 ± 0.03, low Mg^2+^/NMDA F/F_0_: 0.95 ± 0.09, p = 0.58) and total (data not shown) fluorescence intensity in neuronal processes remains unaltered at t = 20 min (Fig.4 D,F). This indicates dispersion of surface GABA_A_R upon low Mg^2+^/NMDA. Somatic GABA_A_R fluorescence intensity is significantly decreased at t = 10 min after low Mg^2+^/NMDA

(control F/F_0_: 1.00 ± 0.02, low Mg^2+^/NMDA F/F_0_: 0.77 ± 0.059, p < 0.01) treatment, however it is not significantly altered at t = 20 min (control F/F_0_: 0.98 ± 0.03, low Mg^2+^/NMDA F/F_0_: 0.85 ± 0.11, p = 0.34) (Fig.4 E). Although the decrease in somatic surface GABA_A_Rs fluorescence intensity during low Mg^2+^/NMDA also occurs rapidly after treatment and is of similar size compared to low Mg^2+^ only treatment, the biphasic recovery phase is shorter in the low Mg^2+^/NMDA treatment.


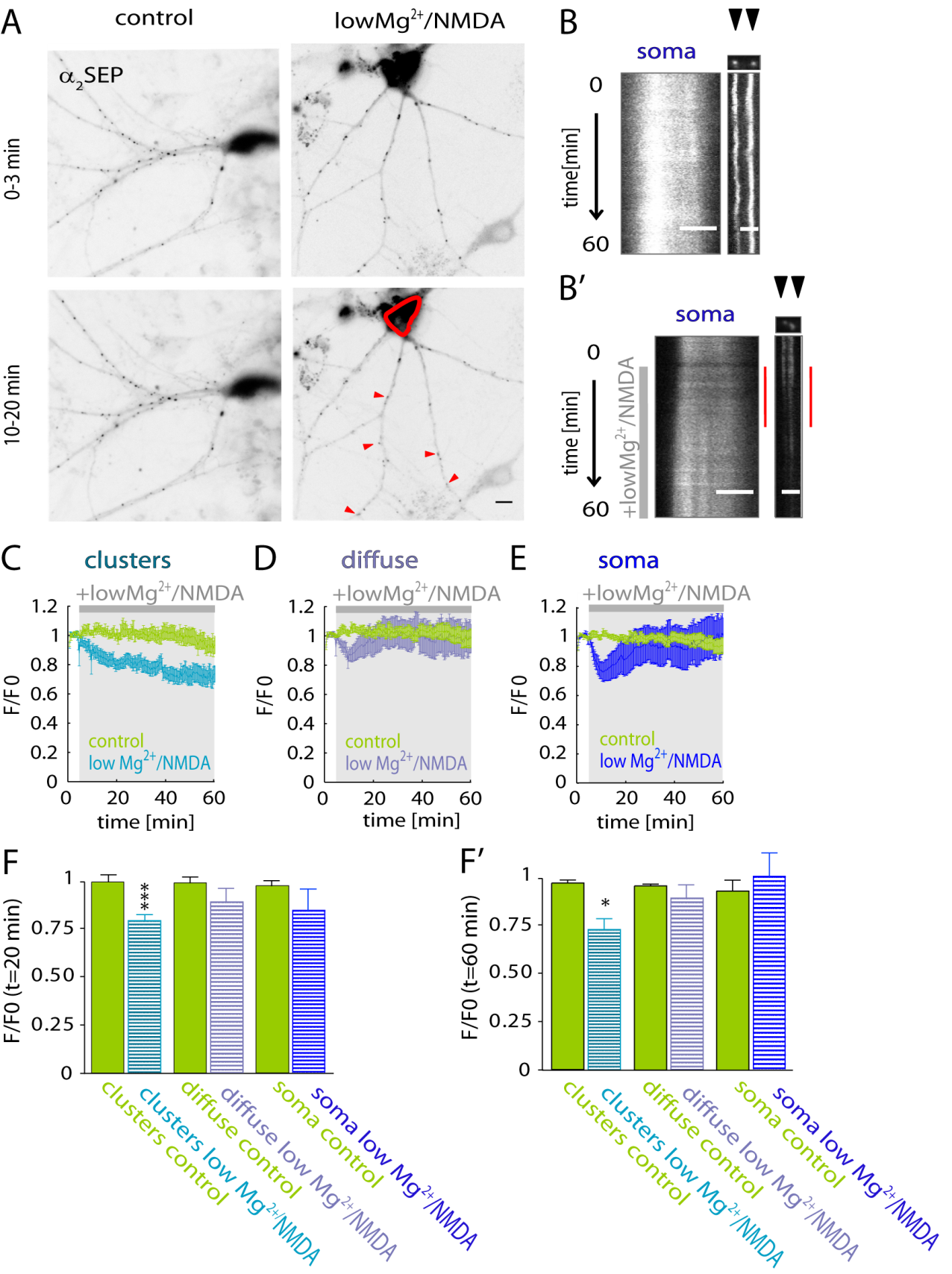


**Figure 4:** Clustered α_2SEP_ GABA_A_Rs decrease upon low Mg^2+^/NMDA treatment. (A) Representative average intensity projection of α_2SEP_ GABA_A_R fluorescence in control and low Mg^2+^/NMDA treated neurons over time (0-3 min and 10-20 min). Somatic α_2SEP_ GABA_A_R fluorescence highlighted in red; scale bar, 10 μm. (B) Kymograph showing somatic (left; scale bar, 5 μm) and clustered (right; scale bar, 2μm) α_2SEP_ GABA_A_R fluorescence intensity over the movie in control (aCSF) conditions and in the presence of low Mg^2+^/NMDA (grey bar). Red bar on the right indicates decrease in somatic α_2SEP_ GABA_A_R fluorescence intensity upon low Mg^2+^/NMDA treatment. (C) Average fluorescence intensity time course of clustered α_2SEP_ GABA_A_R F/F0: control (green, n = 7 cells) and low Mg^2+^/NMDA, (light blue, n = 9). (D) Time course of diffuse α_2SEP_ GABA_A_R F/F0: control (green, n = 7 cells); low Mg^2+^, (magenta, n = 9). (E) Time course of somatic α_2SEP_ GABA_A_R F/F0: control (green, n = 7 cells) and low Mg^2+^ (blue, n = 9). (F) Bar graph of ROI’s F/F0: clusters (left), diffuse (middle) soma (right). Significant loss of fluorescence in the clusters compared to control at 20 min following after low Mg^2+^ treatment (p = 0.0008). Diffuse fluorescence is not altered upon low Mg^2+^ treatment at 20 min (p = 0.36) after low Mg^2+^ treatment. Diffuse fluorescence is unaltered following low Mg^2+^ treatment (t = 20 min; p = 0.58) compared to control. (F’) At 60 after low Mg^2+^ treatment clustered fluorescence intensity is still significantly reduced (p < 0.001), diffuse (p = 0.99) and somatic (p = 0.63) fluorescence are not significantly altered. *p < 0.05, ***p< 0.001.

- 1. **Calcineurin Mediates the Decrease of GABA_A_ Receptors from the Surface during Low Mg^2+^ induced Bursting Activity.**

We next investigated the signalling mechanisms involved in NMDAR mediated GABA_A_R surface decrease during epileptiform bursting activity. Calcineurin is implicated in activity-dependent regulation of GABAergic inhibition and hence could play an important role in Ca^2+^ mediated signalling, we therefore analysed its role in GABA_A_R stability during low Mg^2+^ bursting (Lu et al. 2000; Wang et al. 2003; Chen & Wong 1995; Muir et al. 2010). Cells undergoing epileptiform activity showed a decrease in somatic GABA_A_R fluoresecence intensity compared to control. We found that treating cells with a calcineurin autoinhibitory peptide did not significantly affect somatic GABA_A_R intensity (control F/F0: 1.07 ± 0.10, control/CAIP F/F_0_: 0.95 ± 0.03; p > 0.05, one-way ANOVA test and Tukey’s multiple comparison post test) showing that calcineurin had no effect at t = 20 min in control conditions (Fig.5 C,C’). However, blocking calcineurin activity, inhibited the low Mg^2+^-induced decrease of surface GABA_A_R at the soma at t = 20 min significantly (low Mg^2+^ F/F_0_: 0.75 ± 0.06, low Mg^2+^/CAIP F/F_0_: 1.043 ± 0.06; p < 0.05, one-way ANOVA test and Tukey’s multiple comparison post test) (Fig.5 C, C’). These results suggest that calcineurin activation upon Ca^2+^ influx through NMDARs is directly involved in the decrease of surface GABA_A_R
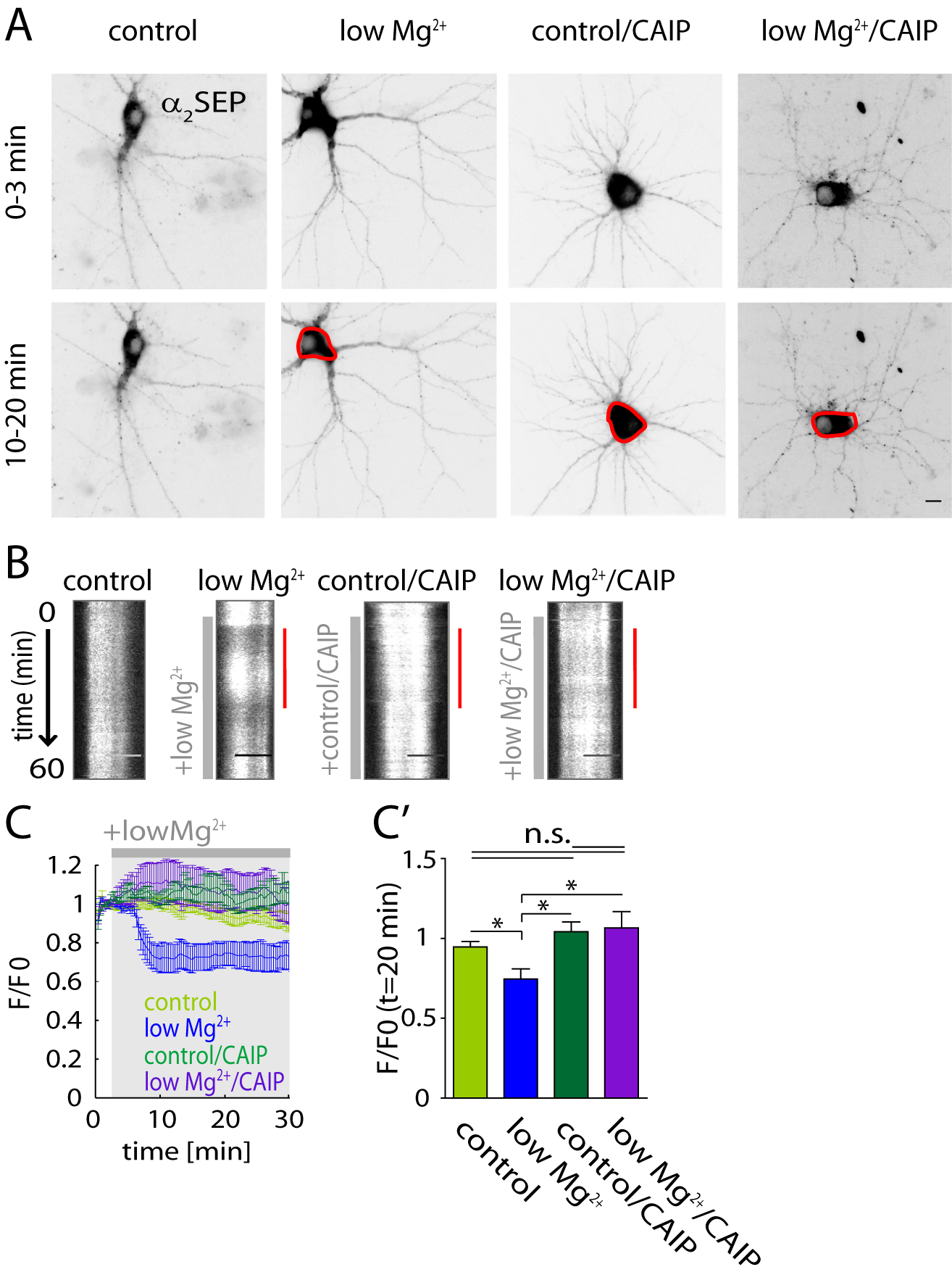
triggered by epileptiform bursting activity.

**Figure 5:** Calcineurin mediates the decrease of somatic surface GABA_A_Rs during low Mg^2+^ treatment. (A) Representative images of α_2SEP_ GABA_A_R fluorescence in control, control with CAIP (control/CAIP), low Mg^2+^ and low Mg^2+^ with CAIP (low Mg^2+^/CAIP) treated neurons as an average intensity projection over time (0-3 min and 10-20 min). Scale bar, 10 μm. (B) Kymographs showing somatic (scale bar: 5 μm) fluorescence intensity over the movie (duration: 30 min) in control conditions, control/CAIP and in the presence of low Mg^2+^ and low Mg^2+^/CAIP (grey bar). Red bar on the right indicates decrease in somatic fluorescence intensity upon low Mg^2+^ treatment. (C) Average fluorescence intensity of α_2SEP_ GABA_A_R F/F0: control (light green, n = 6 cells); low Mg^2+^ (dark blue, n = 7 cells); control/CAIP (magenta, dark green, n = 6); low Mg^2+^/CAIP (n = 6). (C’) Bargraph showing quantification of α_2SEP_ GABA_A_R F/F0 at t = 20 min. Somatic fluorescence intensity of low Mg^2+^ treated cells is significantly decreased compared to control at t = 20 min (dark blue bar, p < 0.05). Treatment of low Mg^2+^ perfused cells with a calcineurin autoinhibitory peptide prevents the change in fluorescence intensity (dark green bar, p < 0.05). Treatment with calcineurin autoinhibitory peptide alone does not significantly alter the fluorescence intensity of GABA_A_R α_2SEP_ (magenta bar, p > 0.05). *p < 0.05

1. **Discussion**

GABA_A_Rs are a target for a variety of drugs including benzodiazepines, which are of high clinical relevance for first-line treatment of SE. Therefore it is likely that the modulation of surface stability of GABA_A_Rs induces or supports benzodiazepine pharmacoresistance in patients with SE. To identify potential mechanisms facilitating GABA_A_R internalisation, we performed live-imaging on SEP-tagged GABA_A_Rs. The key finding of this study shows epileptiform activity induces activation of calcineurin leading to a decrease in the number of surface GABA_A_Rs in the soma. This activity-dependent alteration of inhibitory strength is mediated by activation of NMDARs and is paralleled by an increase in intracellular Ca^2+^ which in turn is likely to activate calcineurin. These data identify a signalling mechanism underlying surface GABA_A_R decrease during SE and supports studies that report NMDAR activation regulates GABAergic inhibition (Muir et al. 2010; Bannai et al. 2009; Stelzer et al. 1987).

It is known that internalisation of GABA_A_Rs containing α, γ_2_ or β_2/3_ subunits is increased during epileptiform activity *in vitro* using low Mg^2+^ or high KCl media (Blair et al. 2004; Goodkin et al. 2008; Goodkin et al. 2005). Furthermore, it has been demonstrated that GABA_A_Rs are internalised in chemoconvulsant models of SE *in vivo* (Naylor et al. 2005; Nishimura et al. 2005). Our data supports the hypothesis that GABA_A_R internalisation occurs during prolonged seizures, by demonstrating that GABA_A_Rs which contain the α_2_ subunits are down modulated during low Mg^2+^ treatment. In the majority of cells this decrease in surface GABA_A_Rs was biphasic possibly indicating an adaptational switch in inhibitory strength. Interestingly, we observe that this effect occurs preferentially in the soma but less in dendrites. Compartmental internalisation of GABA_A_Rs during SE has not been reported and the mechanisms underlying this differential regulation are unknown, but there are a number of possibilities. Firstly, it remains to be investigated to what extent intracellular Ca^2+^ buffering systems such as endoplasmic reticulum or mitochondria are contributing to surface stability of GABA_A_Rs in neurons undergoing epileptiform activity. Expression of signalling proteins in specific subcellular compartments could explain this effect. Secondly, since this study makes use of experiments based on overexpression of GABA_A_Rs, this could account for increased inhibition which could suppress a decrease of GABA_A_Rs in dendrites specifically. Omitting Mg^2+^ in the extracellular medium of cultured hippocampal neurons triggers a sequence of events leading to neuronal death (Kovac et al. 2012; Yoon et al. 2010). To significantly reduce neuronal cell death, mitochondrial complex I substrate sodium pyruvate was added in our experiments. Although we control for this substitution, it would be interesting to test whether mitochondrial ATP production affects GABA_A_R trafficking during prolonged seizures. Thirdly, correlating the amount of intracellular ATP to the trafficking of GABA_A_Rs could contribute to the understanding of compartmentalised trafficking of GABA_A_R during low Mg^2+^ treatment.

A comprehensive body of literature describes that low Mg^2+^ induced epileptiform activity is dependent on increased NMDAR activity (Coan & Collingridge 1985; Coan & Collingridge 1987; Tancredi et al. 1990; Albowitz et al. 1997; Westerhoff et al. 1995; Gulyás-Kovács et al. 2002a; Mangan & Kapur 2004), therefore we tested whether the somatic surface GABA_A_R decrease was mediated by NMDAR activity. Our experiments confirm this hypothesis by reporting that the decrease in surface GABA_A_R was blocked by application of NMDAR blocker dAPV. To our knowledge this is the first study showing a direct regulation of GABA_A_Rs by NMDAR activation during low Mg^2+^ induced epileptiform activity. However, whether the same process underlies the regulation of GABA_A_Rs during epileptiform activity induced by different approaches (Goodkin et al. 2008; Laurén et al. 2005) remains to be determined. The observation that NMDAR activation and rises in internal calcium are common to all models of SE (Raza et al. 2004; Rice & DeLorenzo 1998; Mazarati & Wasterlain 1999) would suggest that this is a universal mechanism.

Interestingly, the direct activation of NMDARs with low Mg^2+^ and NMDA induces a potent change in surface stability of GABA_A_R clusters. The mechanisms underlying this modulation need further investigation, however dephosphorylation of GABA_A_R γ2 subunits could play a role (Muir et al. 2010). Our data suggest that potency of NMDAR activation correlates with the extent of GABA_A_R modification, and may explain the synergistic effect of benzodiazepines and NMDAR antagonists in the treatment of SE (Rice & DeLorenzo 1999; Martin & Kapur 2008)

Ca^2+^ influx from extracellular environment through NMDARs can alter the stability of inhibitory neurotransmitter receptors (Bannai et al. 2009; Muir et al. 2010). Bannai et al. show that diffusion dynamics of GABA_A_Rs too are tuned by Ca^2+^ entry from the extracellular space. Indeed, here we report an increase in intracellular Ca^2+^ concentration during low Mg^2+^ treatment. Moreover, Ca^2+^ levels correlate to the temporal dynamics of the low Mg^2+^ induced effect on GABA_A_Rs. This indicates that fast Ca^2+^-signalling could indirectly be altering GABA_A_Rs surface stability. Although technically challenging, it would be of major interest to simultaneously dual record Ca^2+^ dynamics and GABA_A_Rs surface stability to better address the relationship of low Mg^2+^ induced spiking activity and GABA_A_R trafficking on a single cell level.

Surface stability of GABA_A_Rs is regulated by multiple processes which are facilitated by direct or indirect interaction with trafficking proteins (Marsden et al. 2007; Jacob et al. 2008). Our experiments further emphasise the relationship of indirect signalling via intracellular Ca^2+^ sensing proteins to stabilise neurotransmitter receptors. Downstream effects of Ca^2+^ sensing proteins such as calmodulin orchestrate a number of target proteins and can trigger selective effects on surface GABA_A_R stability. It is known that increased phosphorylation of GABA_A_Rs contributes to surface stability (Saliba et al. 2012). Terunuma et al. demonstrated deficits in GABA_A_R phosphorylation during SE mediated by protein kinase C (Terunuma et al. 2008). Opposingly, dephosphorylation induces declustering and increases the diffusion dynamics of GABA_A_Rs (Muir et al. 2010). Calcineurin has been shown to interact with GABA_A_Rs via the γ_2_ subunit and it modulates neuronal inhibition. Interestingly, basal and maximal activity of calcineurin is increased (Kurz et al. 2001) and subcellular distribution is altered (Kurz et al. 2003) in SE *in vivo*. However, it has been poorly investigated whether calcineurin mediates the decrease in surface GABA_A_Rs (Wang et al. 2009). We identify calcineurin as a mediator of the decrease in surface GABA_A_R and therefore, provide a mechanism of inhibitory modulation during SE, and a potential target for therapy. Moreover, this study is the first to demonstrate a decrease in surface GABA_A_Rs by live-imaging in hippocampal neurons during SE. The identification of the underlying trafficking mechanism could account for resistance to benzodiazepines and could have additive effects on duration or frequency of seizure activity. Further research is needed to develop more effective therapeutic strategies against SE to which this study will contribute.

**ACKNOWLEDGEMENTS**

This work was supported by grants from the UK Medical Research Council, the Wellcome Trust to J.T.K. and UCL School of Life and Medical Sciences Grand Challenge Studentships.

**CONFLICT OF INTEREST STATEMENT**

The authors declare that they have no competing financial interests.

**
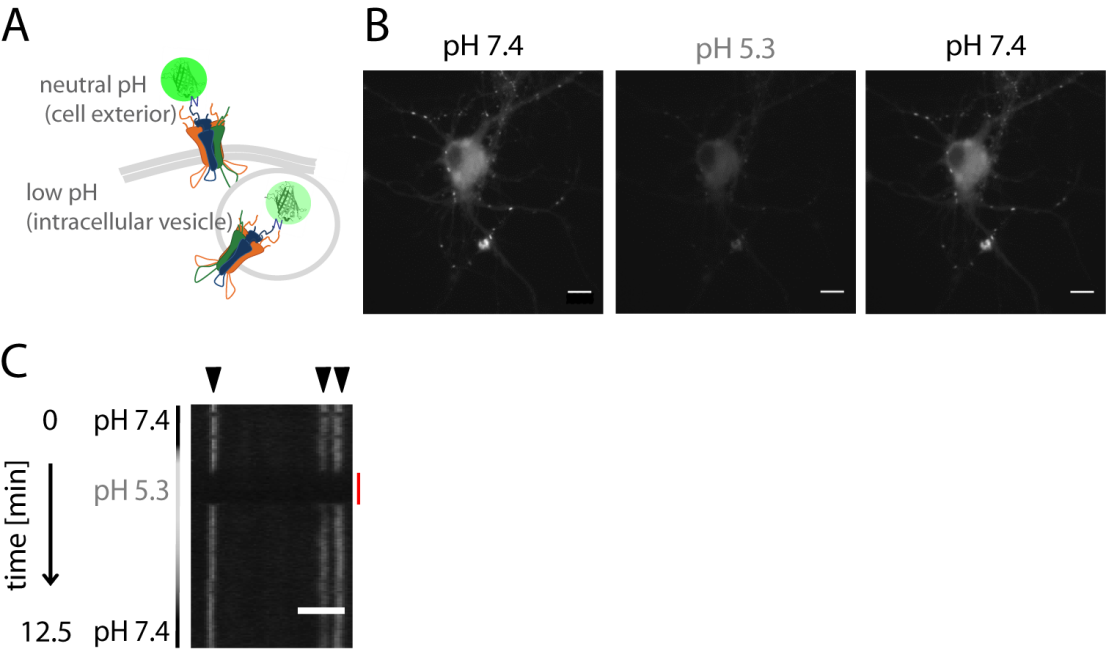
**

**Supplementary Figure 1:** α_2SEP_ clusters are at the cell surface. (A) Schematic illustrating the fluorescence intensity of the N-terminally tagged SEP exposed to extracellular environment and in an intracellular vesicle. (B) α_2SEP_ GABA_A_R fluorescence signal is on the surface. Average fluorescence intensity of α_2SEP_ GABA_A_R transfected hippocampal neurons undergoing pH-dependent fluorescent change. Fluorescence in the soma and dendrites is eclipsed on a transient switch to pH 5.3 solution. Scalebar, 10 µm. (C) Kymograph showing a transient loss of GABA_A_Rs cluster fluorescence during low pH, indicating that fluorescence is from surface GABA_A_Rs. Scalebar, 2 µm.


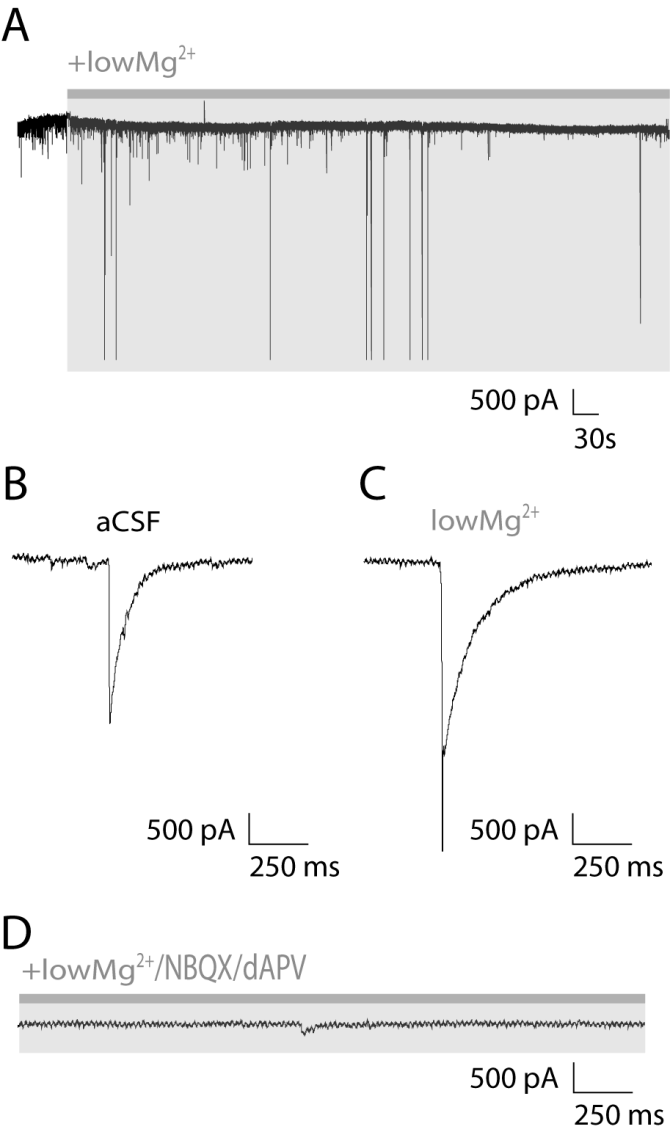


**Supplementary Figure 2:** Application of low Mg^2+^ induces spike activity in GABA_A_R α_2SEP_ transfected cultured hippocampal neurons. (A) Representative voltage-clamp recording in cell-attached mode. Low Mg^2+^ medium induces an increase in detectable post-synaptic currents (PSCs), which are sufficiently large to result in bursts of spike activity. (B) A single PSC during aCSF perfusion and (C) low Mg^2+^ perfusion in the same cell as in (A) results in bursts of larger PSCs with superimposed action potential. (D) Pharmacological sensitivity of low Mg^2+^ induced events was tested by washing in drugs during low Mg^2+^ perfusion. In the same cell as in (A), NBQX/dAPV abolished all PSCs.

1. **References**

Albowitz, B., König, P. & Kuhnt, U., 1997. Spatiotemporal distribution of intracellular calcium transients during epileptiform activity in guinea pig hippocampal slices. *Journal of neurophysiology*, 77(1), pp.491–501.

Bannai, H. et al., 2009. Activity-Dependent Tuning of Inhibitory Neurotransmission Based on GABAAR Diffusion Dynamics. *Neuron*, 62(5), pp.670–682.

Blair, R.E. et al., 2004. Epileptogenesis causes acute and chronic increases in GABAA receptor endocytosis that contributes to the induction and maintenance of seizures in the hippocampal culture model of acquired epilepsy. *The Journal of pharmacology and experimental therapeutics*, 310(3), pp.871–80. Available at: http://www.ncbi.nlm.nih.gov/pubmed/15084648 [Accessed June 11, 2014].

Bogdanov, Y. et al., 2006. Synaptic GABAA receptors are directly recruited from their extrasynaptic counterparts. *The EMBO journal*, 25(18), pp.4381–9. Available at: http://www.pubmedcentral.nih.gov/articlerender.fcgi?artid=1570424&tool=pmcentrez&rendertype=abstract [Accessed May 28, 2014].

Brünig, I. et al., 2002. Intact sorting, targeting, and clustering of gamma-aminobutyric acid A receptor subtypes in hippocampal neurons in vitro. *The Journal of comparative neurology*, 443(1), pp.43–55.

Chen, Q.X. & Wong, R.K., 1995. Suppression of GABAA receptor responses by NMDA application in hippocampal neurones acutely isolated from the adult guinea-pig. *The Journal of physiology*, 482 ( Pt 2, pp.353–362.

Coan, E.J. & Collingridge, G.L., 1987. Characterization of an N-methyl-d-aspartate receptor component of synaptic transmission in rat hippocampal slices. *Neuroscience*, 22(1), pp.1–8. Available at: http://www.sciencedirect.com/science/article/pii/0306452287901928 [Accessed June 15, 2014].

Coan, E.J. & Collingridge, G.L., 1985. Magnesium ions block an N-methyl-d-aspartate receptor-mediated component of synaptic transmission in rat hippocampus. *Neuroscience Letters*, 53(1), pp.21–26. Available at: http://www.sciencedirect.com/science/article/pii/0304394085900916 [Accessed June 15, 2014].

DeLorenzo, R.J., Pal, S. & Sombati, S., 1998. Prolonged activation of the N-methyl-D-aspartate receptor-Ca2+ transduction pathway causes spontaneous recurrent epileptiform discharges in hippocampal neurons in culture. *Proceedings of the National Academy of Sciences of the United States of America*, 95(24), pp.14482–14487.

Dodrill, C.B. & Wilensky, A.J., 1990. Intellectual impairment as an outcome of status epilepticus. *Neurology*, 40(5 Suppl 2), pp.23–7. Available at: http://www.ncbi.nlm.nih.gov/pubmed/2185437 [Accessed June 11, 2014].

Goodkin, H.P. et al., 2008. Subunit-specific trafficking of GABA(A) receptors during status epilepticus. *Journal of Neuroscience*, 28(10), pp.2527–38. Available at: http://www.pubmedcentral.nih.gov/articlerender.fcgi?artid=2880323&tool=pmcentrez&rendertype=abstract.

Goodkin, H.P., Yeh, J.-L. & Kapur, J., 2005. Status epilepticus increases the intracellular accumulation of GABAA receptors. *Journal of Neuroscience*, 25(23), pp.5511–5520. Available at: http://www.pubmedcentral.nih.gov/articlerender.fcgi?artid=2878479&tool=pmcentrez&rendertype=abstract.

Gulyás-Kovács, A. et al., 2002a. Comparison of spontaneous and evoked epileptiform activity in three in vitro epilepsy models. *Brain Research*, 945(2), pp.174–180. Available at: http://www.sciencedirect.com/science/article/pii/S0006899302027518 [Accessed June 15, 2014].

Gulyás-Kovács, A. et al., 2002b. Comparison of spontaneous and evoked epileptiform activity in three in vitro epilepsy models. *Brain research*, 945(2), pp.174–80. Available at: http://www.ncbi.nlm.nih.gov/pubmed/12126879 [Accessed June 15, 2014].

Jacob, T.C., Moss, S.J. & Jurd, R., 2008. REVIEWS GABA A receptor trafficking and its role in the dynamic modulation of neuronal inhibition. *Nature*, 9(MAy), pp.331–343. Available at: http://www.ncbi.nlm.nih.gov/pmc/articles/PMC2709246/.

Kittler, J.T. et al., 2000. Constitutive Endocytosis of GABAA Receptors by an Association with the Adaptin AP2 Complex Modulates Inhibitory Synaptic Currents in Hippocampal Neurons. *J. Neurosci.*, 20(21), pp.7972–7977. Available at: http://www.jneurosci.org/content/20/21/7972.short [Accessed June 11, 2014].

Kittler, J.T. et al., 2004. Huntingtin-associated protein 1 regulates inhibitory synaptic transmission by modulating γ-aminobutyric acid type A receptor membrane trafficking. *Proceedings of the National Academy of Sciences of the United States of America*, 101(34), pp.12736–12741. Available at: http://discovery.ucl.ac.uk/9648/.

Kovac, S. et al., 2012. Prolonged seizure activity impairs mitochondrial bioenergetics and induces cell death. *Journal of cell science*, 125(Pt 7), pp.1796–806. Available at: http://jcs.biologists.org/content/125/7/1796.long [Accessed March 28, 2014].

Kurz, J.E. et al., 2001. A significant increase in both basal and maximal calcineurin activity in the rat pilocarpine model of status epilepticus. *Journal of Neurochemistry*, 78(2), pp.304–315. Available at: http://doi.wiley.com/10.1046/j.1471-4159.2001.00426.x [Accessed June 19, 2014].

Kurz, J.E. et al., 2003. Status epilepticus-induced changes in the subcellular distribution and activity of calcineurin in rat forebrain. *Neurobiology of Disease*, 14(3), pp.483–493.

Laurén, H.B. et al., 2005. Kainic acid-induced status epilepticus alters GABA receptor subunit mRNA and protein expression in the developing rat hippocampus. *Journal of neurochemistry*, 94(5), pp.1384–94. Available at: http://www.ncbi.nlm.nih.gov/pubmed/15992369 [Accessed July 13, 2014].

Lothman, E.W., 1990. The biochemical basis and pathophysiology of status epilepticus. *Neurology*, 40, pp.13–23.

Lu, Y.M. et al., 2000. Calcineurin-Mediated LTD of GABAergic Inhibition Underlies the Increased Excitability of CA1 Neurons Associated with LTP. *Neuron*, 26(1), pp.197–205. Available at: http://www.sciencedirect.com/science/article/pii/S0896627300811502 [Accessed June 15, 2014].

Luscher, B., Fuchs, T. & Kilpatrick, C.L., 2011. GABAA receptor trafficking-mediated plasticity of inhibitory synapses. *Neuron*, 70(3), pp.385–409. Available at: http://www.pubmedcentral.nih.gov/articlerender.fcgi?artid=3093971&tool=pmcentrez&rendertype=abstract [Accessed May 24, 2014].

Mangan, P.S. & Kapur, J., 2004. Factors underlying bursting behavior in a network of cultured hippocampal neurons exposed to zero magnesium. *Journal of Neurophysiology*, 91(2), pp.946–957. Available at: http://www.pubmedcentral.nih.gov/articlerender.fcgi?artid=2892720&tool=pmcentrez&rendertype=abstract.

Marsden, K.C. et al., 2007. NMDA receptor activation potentiates inhibitory transmission through GABA receptor-associated protein-dependent exocytosis of GABA(A) receptors. *The Journal of neuroscience : the official journal of the Society for Neuroscience*, 27(52), pp.14326–37. Available at: http://www.ncbi.nlm.nih.gov/pubmed/18160640 [Accessed June 5, 2014].

Martin, B.S. & Kapur, J., 2008. A combination of ketamine and diazepam synergistically controls refractory status epilepticus induced by cholinergic stimulation. *Epilepsia*, 49(2), pp.248–55. Available at: http://www.pubmedcentral.nih.gov/articlerender.fcgi?artid=2844443&tool=pmcentrez&rendertype=abstract [Accessed July 13, 2014].

Mazarati, A.M. & Wasterlain, C.G., 1999. N-methyl-d-asparate receptor antagonists abolish the maintenance phase of self-sustaining status epilepticus in rat. *Neuroscience Letters*, 265(3), pp.187–190. Available at: http://www.sciencedirect.com/science/article/pii/S0304394099002384 [Accessed July 13, 2014].

Muir, J. et al., 2010. NMDA receptors regulate GABAA receptor lateral mobility and clustering at inhibitory synapses through serine 327 on the γ2 subunit. *Proceedings of the National Academy of Sciences of the United States of America*, 107(38), pp.16679–16684. Available at: http://discovery.ucl.ac.uk/168536/.

Mukherjee, J. et al., 2011. The Residence Time of GABAARs at Inhibitory Synapses Is Determined by Direct Binding of the Receptor  1 Subunit to Gephyrin. *Journal of Neuroscience*, 31(41), pp.14677–14687.

Naylor, D.E., Liu, H. & Wasterlain, C.G., 2005. Trafficking of GABA(A) receptors, loss of inhibition, and a mechanism for pharmacoresistance in status epilepticus. *Journal of Neuroscience*, 25(34), pp.7724–7733. Available at: http://www.ncbi.nlm.nih.gov/pubmed/16120773.

Nishimura, T. et al., 2005. Altered expression of GABA(A) and GABA(B) receptor subunit mRNAs in the hippocampus after kindling and electrically induced status epilepticus. *Neuroscience*, 134(2), pp.691–704. Available at: http://www.ncbi.nlm.nih.gov/pubmed/15951123.

Panzanelli, P. et al., 2011. Distinct mechanisms regulate GABAA receptor and gephyrin clustering at perisomatic and axo-axonic synapses on CA1 pyramidal cells. *The Journal of physiology*, 589(20), pp.4959–80. Available at: http://www.pubmedcentral.nih.gov/articlerender.fcgi?artid=3224886&tool=pmcentrez&rendertype=abstract.

Petrini, E.M. et al., 2014. Synaptic recruitment of gephyrin regulates surface GABAA receptor dynamics for the expression of inhibitory LTP. *Nature communications*, 5, p.3921. Available at: http://www.ncbi.nlm.nih.gov/pubmed/24894704.

Pritchett, D.B. et al., 1989. Importance of a novel GABAA receptor subunit for benzodiazepine pharmacology. *Nature*, 338(6216), pp.582–5. Available at: http://dx.doi.org/10.1038/338582a0 [Accessed May 30, 2014].

Raza, M. et al., 2004. Evidence that injury-induced changes in hippocampal neuronal calcium dynamics during epileptogenesis cause acquired epilepsy. *Proceedings of the National Academy of Sciences of the United States of America*, 101(50), pp.17522–7. Available at: http://www.pubmedcentral.nih.gov/articlerender.fcgi?artid=535000&tool=pmcentrez&rendertype=abstract [Accessed July 13, 2014].

Rice, A.C. & DeLorenzo, R.J., 1998. NMDA receptor activation during status epilepticus is required for the development of epilepsy. *Brain research*, 782(1-2), pp.240–7. Available at: http://www.ncbi.nlm.nih.gov/pubmed/9519269 [Accessed July 13, 2014].

Rice, A.C. & DeLorenzo, R.J., 1999. N-methyl-D-aspartate receptor activation regulates refractoriness of status epilepticus to diazepam. *Neuroscience*, 93(1), pp.117–23. Available at: http://www.ncbi.nlm.nih.gov/pubmed/10430476 [Accessed July 13, 2014].

Robinson, H.P. et al., 1993. Periodic synchronized bursting and intracellular calcium transients elicited by low magnesium in cultured cortical neurons. *Journal of neurophysiology*, 70(4), pp.1606–16. Available at: http://jn.physiology.org/content/70/4/1606.abstract [Accessed May 14, 2014].

Saliba, R.S., Kretschmannova, K. & Moss, S.J., 2012. Activity-dependent phosphorylation of GABAA receptors regulates receptor insertion and tonic current. *The EMBO Journal*, 31(13), pp.2937–51. Available at: http://www.ncbi.nlm.nih.gov/pubmed/22531784.

Sombati, S. & Delorenzo, R.J., 1995. Recurrent spontaneous seizure activity in hippocampal neuronal networks in culture. *Journal of neurophysiology*, 73(4), pp.1706–1711.

Stelzer, A., Slater, N.T. & ten Bruggencate, G., 1987. Activation of NMDA receptors blocks GABAergic inhibition in an in vitro model of epilepsy. *Nature*, 326(6114), pp.698–701. Available at: http://dx.doi.org/10.1038/326698a0 [Accessed July 5, 2014].

Sutter, R. et al., 2013. Mortality and recovery from refractory status epilepticus in the intensive care unit: a 7-year observational study. *Epilepsia*, 54(3), pp.502–11. Available at: http://www.ncbi.nlm.nih.gov/pubmed/23294049 [Accessed May 28, 2014].

Tancredi, V. et al., 1990. Low magnesium epileptogenesis in the rat hippocampal slice: electrophysiological and pharmacological features. *Brain research*, 511(2), pp.280–290.

Terada, H. et al., 2003. Inhibition of excitatory neuronal cell death by cell-permeable calcineurin autoinhibitory peptide. *Journal of neurochemistry*, 87(5), pp.1145–1151.

Terunuma, M. et al., 2008. Deficits in phosphorylation of GABA(A) receptors by intimately associated protein kinase C activity underlie compromised synaptic inhibition during status epilepticus. *Journal of Neuroscience*, 28(2), pp.376–384. Available at: http://discovery.ucl.ac.uk/134982/.

Tretter, V. et al., 2008. The clustering of GABA(A) receptor subtypes at inhibitory synapses is facilitated via the direct binding of receptor alpha 2 subunits to gephyrin. *Journal of Neuroscience*, 28(6), pp.1356–65. Available at: http://www.ncbi.nlm.nih.gov/pubmed/18256255.

Trinka, E., Höfler, J. & Zerbs, A., 2012. Causes of status epilepticus. *Epilepsia*, 53(SUPPL. 4), pp.127–138.

Wang, A. et al., 2009. Calcineurin-mediated GABAA receptor dephosphorylation in rats after kainic acid-induced status epilepticus. *Seizure*, 18(7), pp.519–523.

Wang, J. et al., 2003. Interaction of calcineurin and type-A GABA receptor gamma 2 subunits produces long-term depression at CA1 inhibitory synapses. *The Journal of neuroscience : the official journal of the Society for Neuroscience*, 23(3), pp.826–36. Available at: http://www.ncbi.nlm.nih.gov/pubmed/12574411 [Accessed March 27, 2014].

Westerhoff, C.H., Domann, R. & Witte, O.W., 1995. Inhibitory mechanisms in epileptiform activity induced by low magnesium. *Pflugers Arch.*, 430(2), pp.238–245.

Yoon, J.J. et al., 2010. Effect of low Mg2+ and bicuculline on cell survival in hippocampal slice cultures. *The International journal of neuroscience*, 120(12), pp.752–759. Available at: http://www.ncbi.nlm.nih.gov/pubmed/20942591.
